# Galaxy for accessible, reproducible, and collaborative data analyses: 2026 update

**DOI:** 10.1093/nar/gkag469

**Published:** 2026-06-09

**Authors:** The Galaxy Community, Enis Afgan, Najat Amoukou, Rafael Andrade Buono, Mihail Anton, Christophe Antoniewski, Patrick Austin, Ahmed Hamid Awan, Rolf Backofen, Wendi Anne Bacon, Dannon Baker, Arthur Barreau, Bérénice Batut, Matthias Bernt, Daniel Blankenberg, Anthony Bretaudeau, Catherine Joan Bromhead, Richard Burhans, Melissa Burke, Scott Cain, Danielle Callan, Martin Cech, María Chavero-Díez, Haswane Chei, Ying Chen, Diana Chiang, John M Chilton, Tyler James Collins, Frederik Coppens, Nate Coraor, Charles Coulombe, Michael R Crusoe, Fabio Cumbo, Triskell Cumunel, Michael D'Silva, Armin Dadras, Céline Dalle, Khai Dang, John Y Davis, Paul De Geest, Willem de Koning, Giuseppe Defazio, Boris Depoortere, Katherine Do, José Manuel Domínguez Begines, Bert Droesbeke, Erick Nicolas Duarte, Enol Fernández-del-Castillo, Jeremy Fix, Giulio Formenti, Julian Frey, Melanie Christine Föll, Carol Gauthier, Kilian Gerberding, Franck Giacomoni, Jeremy Goecks, Nuwan A Goonasekera, Nadia Goué, Timothy J Griffin, Björn Andreas Grüning, Aysam Guerler, Yann Guitton, Ove Johan Ragnar Gustafsson, Kristína Gömöryová, Magdalena Harakalova, Helge Hecht, Alireza Heidari, Florian Heyl, Jennifer Hillman-Jackson, Saskia Hiltemann, Mina Hojat Ansari, Hans-Rudolf Hotz, Cameron John Hyde, Pierre-Étienne Jacques, Pratik Dilip Jagtap, Jayadev Joshi, Marie Jossé, Khaled Moahmmad Ahmad Jumah, Arash Kadkhodaei Elyaderani, Katarzyna Kamieniecka, Teja Kattenborn, Markus Konkol, Leonid Kostrykin, Natalie Kucher, Anup Kumar, Mira Kuntz, Bradley William Langhorst, Delphine Lariviere, Yvan Le Bras, Gildas Le Corguillé, Jean Le Cras, Justin Lee, Jan Leendertse, Hugo Lefeuvre, Brane Leskošek, Leandro Miguel Liborio, Romane Libouban, Marisa Loach, Jose David Lopez Tabernero, Lucille Lopez-Delisle, Daniel Lusk, Alexandru Mahmoud, Molène Mahé, Wolfgang Maier, Igor Makunin, Kirsty McCaffrey, Jack A Medico, Subina Mehta, Hailiang Mei, Mirela Minkova, Saim Momin, Paulo Cilas Morais Lyra Junior, Teresa Müller, Amirhossein Naghsh Nilchi, Tannistha Nandi, Engy Mohamed Taha Nasr, Anton Nekrutenko, Tiffanie Nelson, Johannes Nussbaum, Asime James Oba, Łukasz Opioła, Kevin Payet, Melanie Petera, Polina V Polunina, Sergei Pond, Krzysztof Poterlowicz, Gareth Robert Price, Junhao Qiu, Helena Rasche, Bryan Raubenolt, Tristan N Reynolds, Dave Rogers, Karl Rohr, Gabriel Ferreira Saudade, Michelle Terese Savage, Volodymyr Savchenko, Michael C Schatz, Isabelle Schmitz, Daniela Schneider, Pauline Seguineau, Beatriz Serrano-Solano, Clea Siguret, Patrik Smeds, Marco Sollitto, Nicola Soranzo, Sanjay Kumar Srikakulam, Lieven Sterck, Nikolaos Strepis, Andrew Stubbs, Keith Suderman, Anna Syme, Marco Antonio Tangaro, Reyhaneh Tavakoli Koopaei, Jonathan Andrew Tedds, Mehmet Tekman, Wai Cheng (Mike) Thang, Anil S Thanki, Michael Uhl, Janusch Vajna-Jehle, Marius van den Beek, Deepti Varshney, Nikolay Alexandrov Vazov, Jennifer Vessio, Pavankumar Videm, Tomas Vondrak, Reid Wagner, Gregory R Watson, Ralf J M Weber, Natalie Whitaker-Allen, Federico Zambelli, Paul Zierep, Rand Zoabi

## Abstract

Galaxy (https://galaxyproject.org), now in its third decade, is a globally accessible, community-driven platform for reproducible and collaborative data analysis. With over 650 000 registered users, major public servers handle ~2 000 000 monthly analysis jobs from ~20 000 users. Recent, massive modernization has overhauled the user interface and workflow infrastructure to improve usability and scalability. Key updates include a redesigned history interface, enhanced tool discovery, a centralized dataset view, and a new visualization framework. Workflow editing is now substantially enhanced with search, undo/redo, and new invocation graph views. Data management is more flexible with intelligent sample sheet handling, expanded collection support, redesigned file source interfaces, and the introduction of “scratch” storage. User-defined repositories like Google Drive, Dropbox, and Zenodo are now enabled by default. Coupled with the growth of the Galaxy Training Network, these advances solidify Galaxy as a robust, widely adopted AI-ready ecosystem for open science.

## Graphical Abstract

**Graphical Abstract:**
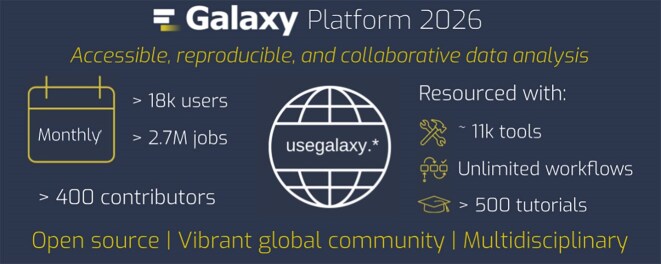

## Introduction

Data-intensive research continues to expand in both scale and complexity across biomedical, environmental, and computational sciences. The increasing reliance on high-throughput technologies, large reference datasets, heterogeneous analysis tools, and scalable computing infrastructure presents ongoing challenges for accessibility, reproducibility, and transparency. Since its introduction in 2005 through today [1, 2], Galaxy has addressed these challenges by providing an open, web-based platform that allows researchers to perform complex computational analyses.

The Galaxy ecosystem comprises an extensible software framework, a global federation of public and institutional servers, a shared tool and tool container distribution infrastructure, integrated reference datasets, and an active international community of developers and educators. Major public Galaxy servers, including https://usegalaxy.org, https://usegalaxy.eu, https://usegalaxy.org.au, and nation-level UseGalaxy instances, collectively support >650 000 registered users with a monthly average of nearly 20 000 active users running 2 000 000 jobs. These servers are connected to national computing infrastructures, enabling multicore, high-memory, and GPU-intensive analyses accessible to users through a web browser without local installation or licensing barriers. All Galaxy instances have access to nearly 11 000 integrated tools spanning genomics, proteomics, metabolomics, imaging, cheminformatics, ecology, earth sciences, humanities, and related domains.

The 2024 update [3] emphasized user experience design improvements, workflow support for global research consortia, environmental impact reporting, and enhanced storage visibility. The 2026 update builds upon these foundations with a comprehensive modernization of the user interface, substantial improvements to workflow authoring and inspection, expanded data management flexibility, including scratch storage and default user-defined repositories, and a transition to a modern visualization architecture.

## New user experience

Over the past two years, Galaxy has undergone a broad user interface refresh designed to improve clarity, scalability, and consistency across core components. These changes reflect accumulated community feedback and the growing complexity of analyses conducted within Galaxy. Underpinning these improvements is a completed transition to a single-page application architecture with client-side routing, eliminating full-page reloads during navigation and enabling a more responsive, application-like experience throughout the platform.

### Activity Bar and navigation redesign

**Figure 1.**
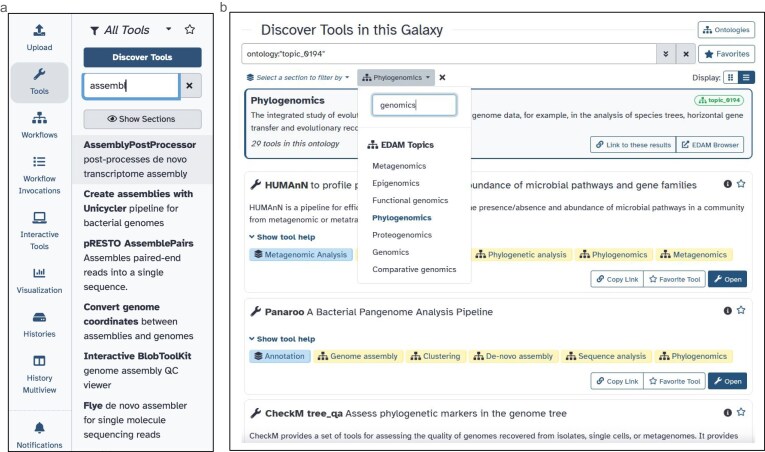
(**a**) Screenshot of the Activity Bar on the left showing multiple tabs and the tool discovery panel showing the dynamically generated results shown when “assembl” is typed in the search bar. (**b**) The Discover Tools view showing the results searching for phylogenomics tools.

Galaxy’s interface layout has been reorganized around a new Activity Bar (Fig. 1a), replacing the previous masthead-centric navigation. The Activity Bar provides persistent access to histories, workflows, visualizations, interactive tools, and notifications, while the masthead has been consolidated to reduce redundancy. A dedicated Interactive Tool panel allows users to monitor active sessions and manage interactive environments directly from the Activity Bar.

#### Enhanced tool discovery

Tool discovery has evolved from primarily keyword-based search to a more structured browsing experience. The redesigned discovery interface supports improved filtering by name, identifier, help text, and section, alongside clearer presentation of tool metadata (Fig. 1b). A new EDAM ontology [4] view allows users to browse tools grouped by annotated topic and operation types, and a card-based layout supports quick scanning of tool descriptions and documentation. Example input and output data are now available within each tool form, supporting self-guided tool configuration and reducing errors from misconfigured inputs or outputs. These improvements streamline navigation across the thousands of tools available on major Galaxy services.

#### Unified dataset view

**Figure 2.**
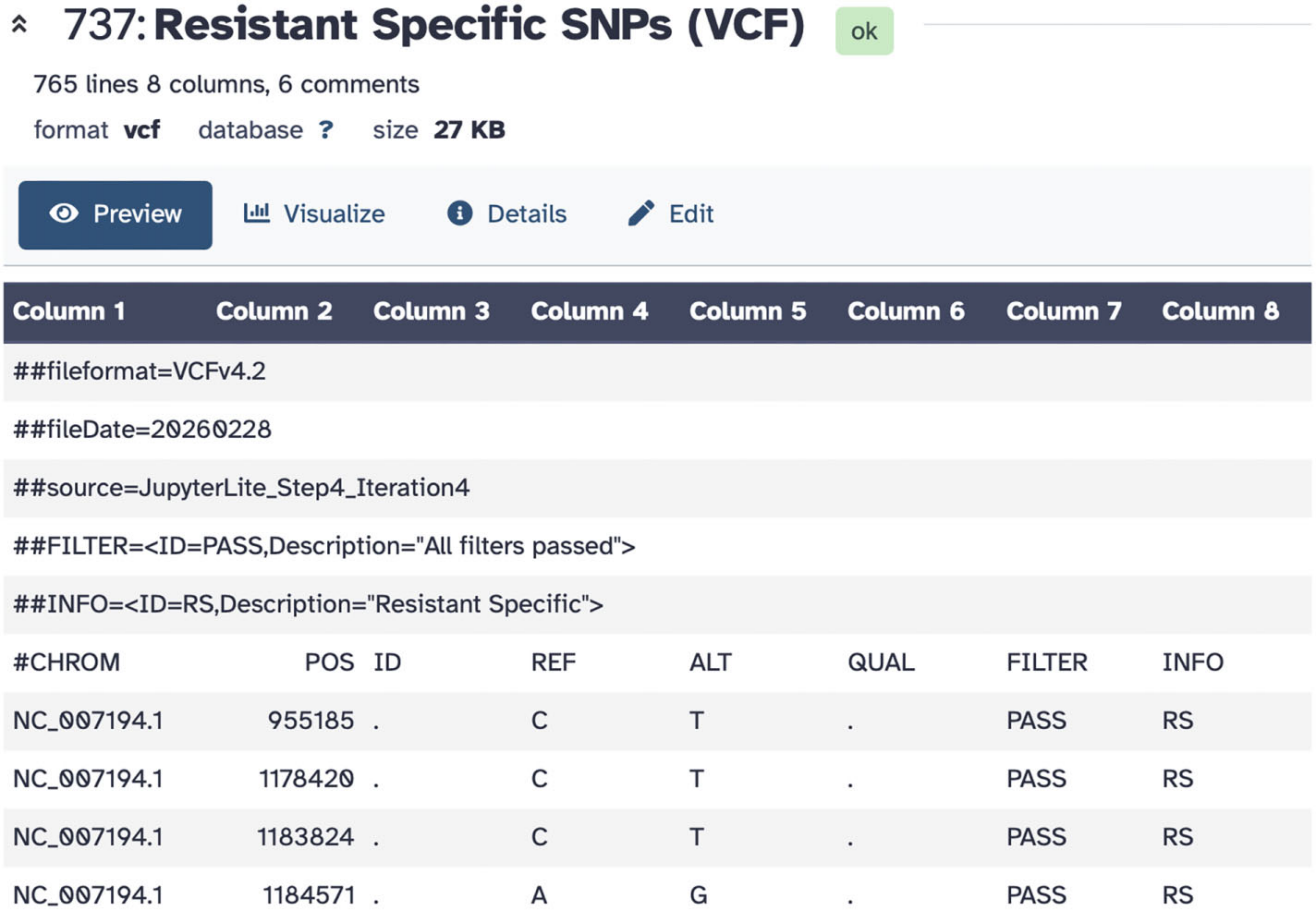
Screenshot of a dataset view of a generated VCF file, with Preview, Visualize, Details, and Edit tabs.

Dataset interactions have been consolidated into a unified dataset view. Actions previously distributed across multiple views, such as metadata inspection, datatype management, visualization selection, permission configuration, and annotation, are now accessible through a structured tabbed interface instead of several distinct pages (Fig. 2). This unification reduces information fragmentation and establishes consistent interaction patterns across datasets, collections, and derived artifacts. The unified view improves the discoverability of dataset properties and enables tighter integration with the visualization framework, making visualization options easier to find. Administrators can configure default visualizations per datatype, ensuring that binary or specialized formats open directly in appropriate viewers rather than generic previews.

#### Redesigned history list

The history list has been redesigned with a structured, card-based layout that improves readability and scalability when managing large numbers of datasets and analyses. Each history displays key metadata including creation date, update status, dataset count, and storage footprint. Users may toggle between grid and list views depending on preference, and range selection enables efficient bulk operations on history items. Additional refinements include improved filtering, faster navigation, and clearer indicators of dataset states within a history.

#### Additional interface improvements

Keyboard navigation has been introduced throughout the interface, including arrow-key traversal of history items, shift-click range selection, and keyboard shortcuts for common operations. Multi-item drag and drop allows users to move datasets between histories or directly into tool input forms. A live console output view provides real-time display of job standard error and standard output during execution, reducing the need to wait for job completion before diagnosing issues.

Interactive tools are now launched within the central interface panel instead of external browser windows, enabling a more integrated and cohesive user experience. This integration facilitates bidirectional, real-time data exchange between supported interactive tool sessions (e.g. Jupyter Notebook) and the Galaxy history. A dedicated view for recent downloads and exports enhances traceability and makes it easier to locate shared artifacts. The user preferences interface has been restructured with clearer organization of settings related to account management, storage configuration, external repositories, and workflow preferences.

## Workflow management and execution

Workflows remain foundational to Galaxy’s commitment to reproducibility, scalability, and transparency. Workflow authoring, execution, documentation, and inspection capabilities have been substantially modernized in recent years. We have also developed the Intergalactic Workflow Commission (IWC) repository of open, peer-reviewed workflows spanning dozens of topics in genomics, metabolomics, proteomics, imaging, and beyond (iwc.galaxyproject.org) and is integrated with community efforts like WorkflowHub [5] and Dockstore [6] using the GA4GH TRS standard [7]. Relatedly, we have hardened support for running workflows programmatically via the Planemo API and CLI [8] as well as for cloud deployments using Helm charts [9] and through our Pulsar network [10].

### Workflow Editor modernization

The Workflow Editor has been significantly enhanced to support complex graph editing. Search functionality allows users to locate specific steps, tools, or subworkflows within large workflows. Multi-item selection enables users to select, move, duplicate, or delete multiple components simultaneously, reducing friction in graph restructuring.

An undo/redo stack supports iterative development, and the interface provides a visible navigation mechanism for tracking editing history. Subworkflows can be cloned directly within larger workflows, improving modular reuse. Layout improvements and floating inspection panels enhance readability and reduce the need for excessive scrolling in complex graphs.

### Enhanced workflow run form

**Figure 3.**
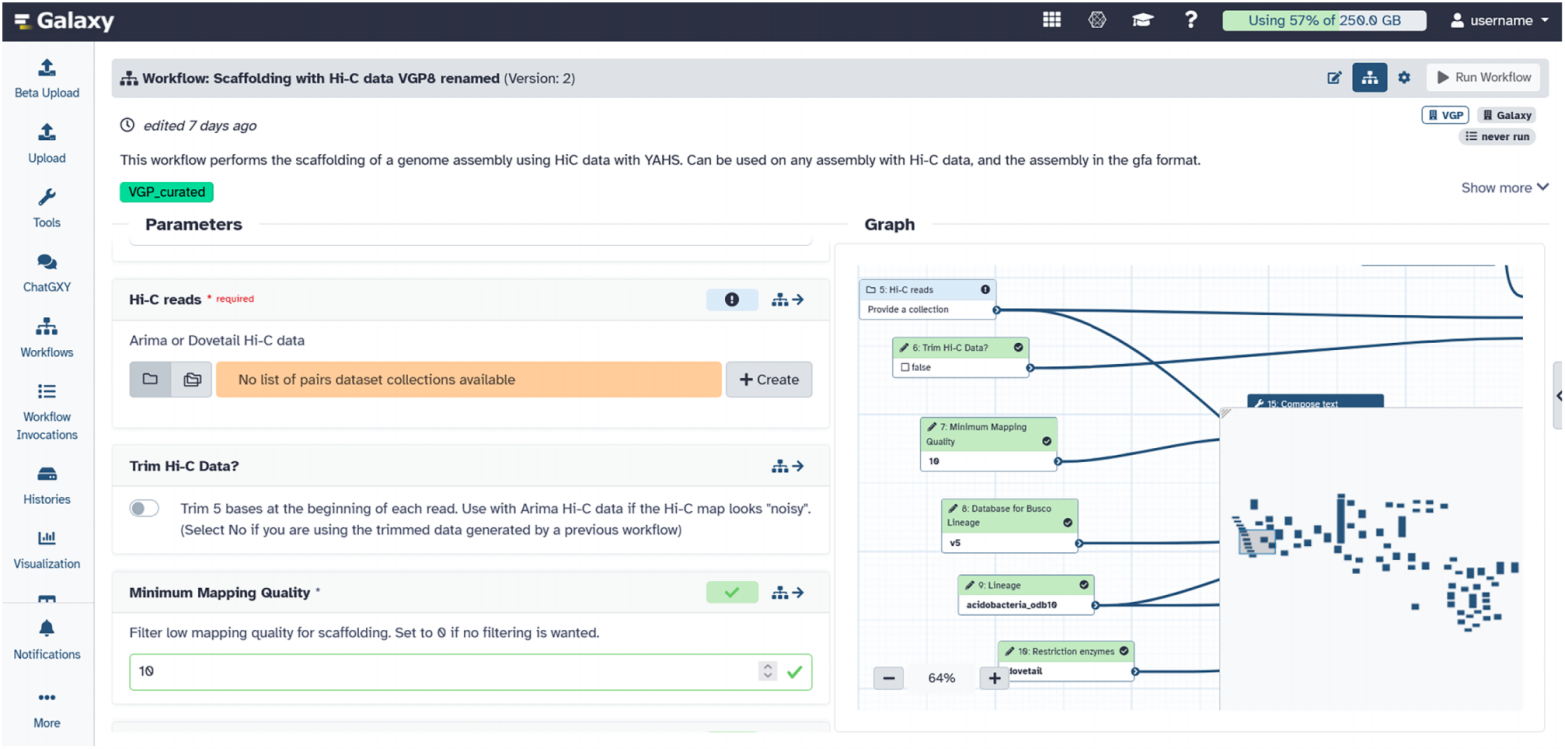
Screenshot of the workflow execution page for the “Scaffolding with Hi-C data VGP6” workflow, showing options in the left panel and a graph of the workflow steps in the right panel.

The workflow run form has been redesigned to provide clearer grouping of parameters and improved mapping of dataset collections (Fig. 3). Users can more easily identify required inputs and adjust configuration settings. New Smart Sample Sheets integrate directly into the run form, enabling structured mapping of tabular metadata to workflow parameters. Workflow authors can attach markdown-formatted README documentation directly to workflow definitions. This documentation may include explanatory text, parameter descriptions, references, and images, and is accessible within the workflow run form, supporting clarity and reducing barriers to reuse.

### Invocation graph visualization

**Figure 4.**
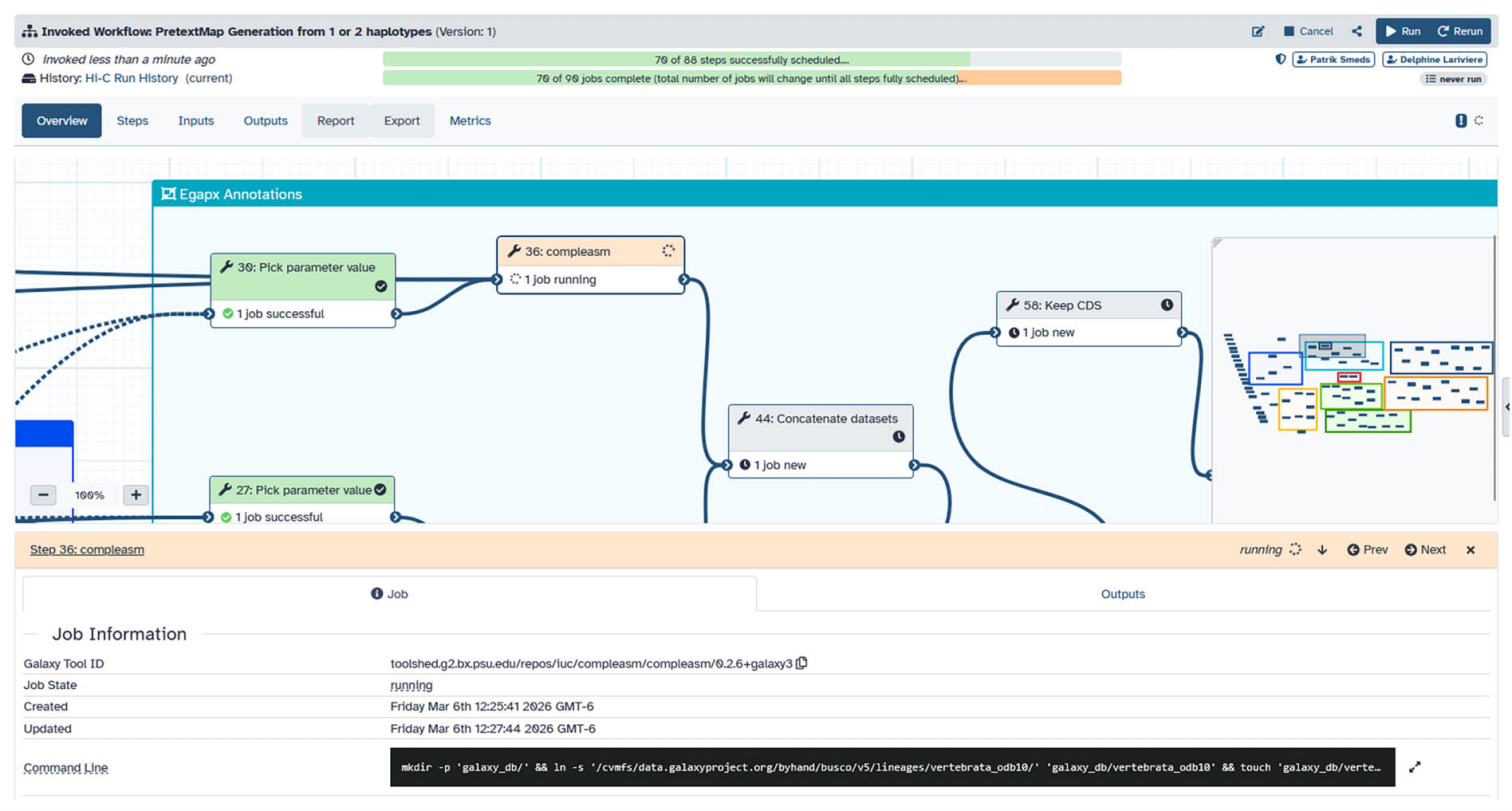
Screenshot of a small portion of the “Hi-C Contact Map Generation for Manual Curation of Genome Assemblies” (https://iwc.galaxyproject.org/workflow/hi-c-contact-map-for-assembly-manual-curation-main/) workflow shown mid-execution (green items are successful, orange are currently running, gray are new).

An alternative view to the Galaxy history is the new Invocation Graph View. This view allows users to visualize workflow execution overlaid on the workflow canvas in real-time (Fig. 4). While the history is a linear view, the Graph Invocation can visualize arbitrary complex branches of workflows. During or after execution, the invocation graph displays step-level status information, including completed, running, failed, or pending states. Users can interact with the graph to inspect step details and navigate to associated datasets.

In addition to steps, execution details are presented in separate tabs. Invocation summaries provide structured access to inputs, outputs, metrics, and the workflow report. Invocation metrics now include runtime characteristics such as execution duration and resource usage (memory, CPUs). These metrics support performance analysis and workflow optimization without requiring access to underlying cluster logs or navigating through hundreds of workflow steps individually.

Export infrastructure has been extended for workflows and invocations, especially for RO-Crates [11]. Reusing the previously integrated wizard-component, an export interface guides users through structured export of workflow invocations and associated artifacts. Export options include standardized formats such as RO-Crate and BioCompute Objects https://en.wikipedia.org/wiki/BioCompute_Object), and plugin-based extensions, optionally triggered after workflow completion, allow integration with external research data management systems, including InvenioRDM-based repositories.

## Data handling and storage

Galaxy’s data handling model has been extended to support increasingly heterogeneous research data structures. Collection handling has been generalized to enable more expressive nested and structured dataset groupings. Collection builders have been redesigned to improve navigation and rule-based mapping to make complex data analysis easier. Advanced collection filtering operations allow workflows to recover partial results by excluding incomplete or failed datasets. In addition, all-versus-all analysis patterns can be supported through cross-join-style collection generation, enabling systematic pairwise comparisons within workflow execution. Importantly, collection operations do not increase storage usage, since they create no new source files and only restructure references to existing datasets within Galaxy histories.

**Figure 5.**
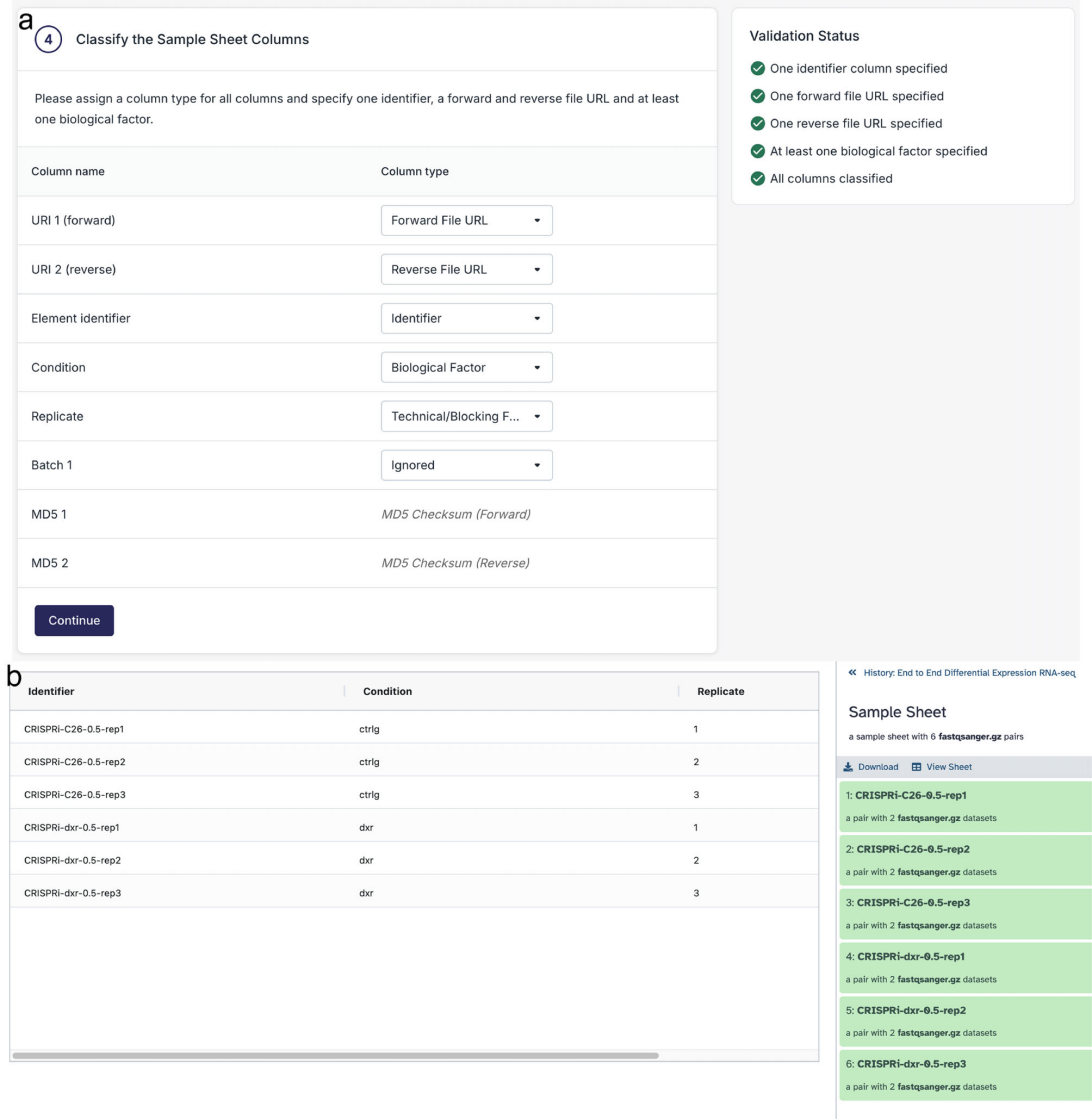
Guided authoring of a sample sheet via BRC-Analytics and its integration with Galaxy. (**a**) Step 4 of the BRC-Analytics guided wizard at brc-analytics.org prompts the user to classify each column of the uploaded sample sheet. Columns are assigned types including forward/reverse file URL, identifier, biological factor, and technical/blocking factor. A live validation panel confirms that all required column types are correctly specified before proceeding. (**b**) The resulting sample sheet is imported directly into a Galaxy history as part of the end-to-end differential expression RNA-seq workflow, where each of the six samples (CRISPRi-C26 and CRISPRi-dxr, three replicates each) is represented as a pair of compressed fastqsanger.gz datasets ready for downstream analysis.

The introduction of Smart Sample Sheets further extends Galaxy’s structured input model by allowing workflow authors to define tabular templates that map sample attributes to workflow parameters. This approach reduces ambiguity in large-cohort studies and enhances reproducibility through explicit metadata-driven mapping. Users can upload sample sheets created in their favorite spreadsheet software or spreadsheets pre-generated by sequencing facilities (Fig. 5a and b). Alternatively, users can interactively fill in sample sheets in a guided wizard, where workflow authors provide help text and type validation to clearly communicate data requirements, such as provided by BRC-Analytics.org, instead of requiring users to provide arbitrarily-shaped files with sample information.

Data ingestion has been improved through the introduction of a ZIP explorer, which enables selective import of individual files from local or remote archives without full extraction. This supports more efficient interaction with archived datasets and remote storage systems and lays the foundation for more powerful RO-Crate imports in the future.

File source interfaces have undergone significant redesign. File systems based on the Python library fsspec are now supported and available file source templates and storage locations are now presented through a card-based layout with breadcrumb navigation and search functionality. Server-side pagination enables responsive navigation of large remote repositories. User-defined file sources are enabled by default on major Galaxy services, allowing users to configure personal or institutional repositories, including Google Drive, Dropbox, OwnCloud, Nextcloud, OMERO, OneData eLabFTW, RSpace, HuggingFace, and Zenodo (via Invenio-based integrations), as navigable file systems within Galaxy. These integrations support direct browsing, selective import, and structured export of datasets without intermediate manual downloads.

Many public Galaxy instances have introduced temporary (“scratch”) storage backends to provide increased flexibility for exploratory and intermediate analyses. Scratch storage allows datasets to be written to short-lived object stores optimized for transient processing, reducing long-term storage pressure while remaining transparently integrated within Galaxy histories. This deployment model complements preferred object store configurations introduced previously and reflects the increasing heterogeneity of storage infrastructure supporting Galaxy services.

Additional storage lifecycle improvements include visual indicators for short-term storage expiration and enhanced tracking of export provenance. Histories that are archived in external repositories display associated document object identifiers, improving clarity and traceability for published analyses.

## Interactive environments and visualization

**Figure 6.**
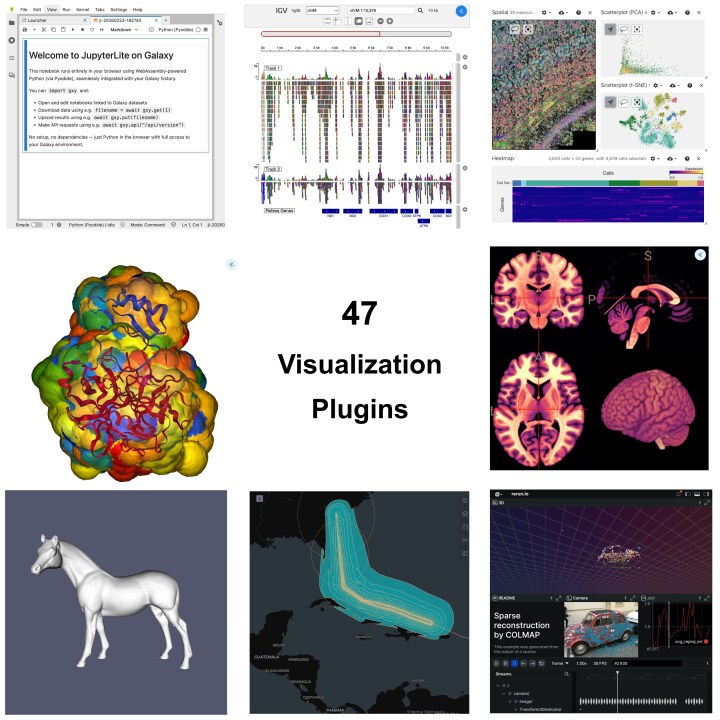
The Galaxy Visualization Framework currently includes 47 plugins. Shown are example screenshots of JupyterLite (https://jupyter.org), IGV.js (https://igv.org), Vitessce (https://vitessce.io/), NGL (https://nglviewer.org), NiiVue (https://niivue.com/), VTK (https://vtk.org/), Kepler.gl, and Rerun.io.

Galaxy has transitioned to a modern visualization architecture through the introduction of the Galaxy Charts Visualization Framework (https://charts.galaxyproject.org). This modular, plugin-based system replaces legacy components and enables improved performance, maintainability, and extensibility. Visualizations are implemented as standalone, versioned JavaScript applications with automated test coverage, which can be installed, developed, and executed independently of Galaxy while integrating seamlessly through the plugin interface. The framework supports a wide range of visualization modalities, including genomic tracks, imaging data, molecular structures, geospatial datasets, alignment viewers, and exploratory plotting tools (Fig. 6).

JupyterLite, a fully browser-based Jupyter environment, is now available as an integrated visualization, including AI support, through admin-configured access, to an LLM. Users can interact with Galaxy datasets and APIs directly within a notebook running in the user’s browser environment without requiring local software installation or containerized interactive tools. This lightweight integration supports exploratory scripting and analysis while maintaining Galaxy’s provenance and data management model.

Galaxy has transitioned to an IGV.js-based genome browser [12], providing a fully browser-native visualization environment that supports a broad range of indexed genomic datatypes without requiring local installation. IGV.js enables dynamic genome selection directly from history-resident FASTA or 2bit datasets and improves interaction with large indexed files through responsive, in-browser rendering. The removal of external desktop dependencies simplifies deployment in training environments, including Galaxy Training Network (GTN) tutorials, where consistent genome browser behavior across participants is essential. In addition, domain-specific viewers contributed by community groups extend visualization support to diverse scientific modalities.

Collectively, Galaxy public services now provide >47 visualization plugins across diverse scientific domains, enabling interactive exploration alongside reproducible pipeline execution while preserving clear versioning, testing, and independent release cycles for each visualization application.

## Integration with external resources

### Workflow and dataset landing pages as integration hooks

Galaxy now provides workflow and dataset landing pages designed as lightweight integration hooks for external services. These landing pages allow third-party platforms, such as data repositories, registries, or domain-specific portals, to redirect users to a Galaxy instance with predefined context, without requiring custom backend infrastructure.

Through parameterized landing page URLs and associated APIs, external websites can initiate Galaxy workflows with prefilled parameters and referenced datasets. Data can be pushed directly into Galaxy and associated with a selected workflow, enabling users to start analysis immediately upon arrival. Because this mechanism operates entirely through URL-based redirection and frontend interactions, it can be implemented even from static websites, lowering the barrier for integration.

This approach allows repositories and registries to expose “Analyze in Galaxy” functionality directly from their own interfaces. Early adopters include the IWC (https://iwc.galaxyproject.org), the BRC-Analytics platform (https://brc-analytics.org) [13], and Dockstore/AnVIL (https://anvilproject.org) [14, 15], which integrate Galaxy workflows into their analytical pipelines via these landing page mechanisms. These integrations demonstrate how Galaxy can function as an execution layer embedded within broader scientific ecosystems, while preserving Galaxy’s reproducibility and provenance model.

By formalizing workflow invocation and dataset ingestion through structured landing pages, Galaxy strengthens interoperability between data repositories and computational analysis environments without requiring complex middleware or service-specific adapters.

### Structured credential management for external service integrations

Galaxy now provides structured support for tools that require external authentication. Tool definitions may declare credential requirements, and users can manage credentials through a dedicated interface separate from workflow definitions. Credential grouping, validation, and management APIs allow users to organize authentication parameters efficiently. Credentials are injected at execution time, avoiding embedding sensitive values directly in workflow objects. As first examples, the community has adopted these new capabilities into tools that upload data to ENA, interact with the Copernicus data archive or the ChatGPT tool.

Similar to BioBlend [16], which provides a Python interface to the Galaxy API, and enabling programmatic access to Galaxy assets within the Python ecosystem, a new R package called GalaxyR [17] is now providing a comprehensive interface to the Galaxy API, allowing users to manage histories, upload data, execute tools and workflows, and retrieve results directly from within R.

## Community and training

Galaxy development remains community-driven, with >400 contributors in the last two years participating in code development, tool integration, documentation, training, and infrastructure operations. Governance structures, including the Galaxy Executive Board, Technical Board, and Community Board, continue to evolve to support a growing international initiative (https://galaxyproject.org/community/governance/). Community engagement spans multiple communication platforms and regional special interest groups, reflecting Galaxy’s commitment to inclusive and transparent governance. This engagement is further strengthened by the Galaxy Codex (https://training.galaxyproject.org/training-material/topics/community/faqs/create_codex.html), a collaborative space where communities can collect, curate and expose their tools, workflows, and tutorials, and the Galaxy Labs [18].

In the last two years, communities such as imaging, microbiology, single-cell, biodiversity, and earth system sciences have used these resources to provide users with state-of-the-art, community-curated content within the Galaxy environment [19–22]. Galaxy also serves as a primary analysis platform for several major initiatives, including the Vertebrate Genomes Project [23] and the BioDIGS soil metagenomics project [24]. Additionally, over 30 communities of interest, generating datasets of national significance through BioPlatforms Australia Initiatives, have all transitioned to Galaxy as the primary analytical tool for newly generated data (https://bioplatforms.com/initiatives/). Moreover, Galaxy continues to advance into new scientific fields, such as the Digital Humanities and Social Sciences [25].

The Galaxy Community Hub (http://galaxyproject.org) was redesigned to renew the underlying infrastructure, simplify contributions, provide a more modern look and feel and adopt structured metadata shared with the GTN [26, 27]. This unified metadata framework improves discoverability of tagged posts, events, contributors, organizations, and funders, providing a clearer and more comprehensive overview of community activity across the Galaxy ecosystem. The Hub has published ~1700 news items and documented 1370 community events, illustrating the breadth and vitality of the project’s global engagement.

The GTN (https://training.galaxyproject.org/) now provides over 500 self-paced tutorials across 16 scientific domains. Learning pathways and direct tool-linked training materials reduce barriers for new users and support global research consortia. Leveraging this broad topical coverage, GTN offers an annual global massive open online course training event called the Galaxy Training Academy (GTA). Completely free, they include introductions to the Galaxy ecosystem, and in-depth “tracks” for specific research subjects such as machine learning or genome assembly. At the date of this paper’s writing, the third instance of this event, GTA2026, is being organized. These week-long sessions are truly global: slack communication channels are staffed around the clock by members of the US, European, and Australian Galaxy communities. GTA2024 recorded over 2800 registered participants and covered 9 topics, while GTA2025 recorded over 3500 registered participants and 10 topics. They are a large community effort, with a minimum of 70 people involved at some level in the creation of the materials and the organization of the events.

In addition to technical and training activities, the Galaxy community has continued to reflect on the long-term sustainability of the project itself, including governance, funding, maintenance capacity, and resilience to changes in personnel and infrastructure providers. To support transparency and community planning, the project has produced a Sustainability Report (https://gxy.io/sustainability) that summarizes how Galaxy is sustained as a global open-source effort, discusses contributor distribution and “bus factor” considerations, and outlines approaches for strengthening long-term support through diversified funding and community engagement. This report provides a shared baseline for assessing project health and guiding future investment in Galaxy as critical research infrastructure.

We also completed a multiyear transition of the Galaxy codebase from the Academic Free License 3.0 (AFL-3.0) to the MIT License. Galaxy originally adopted the AFL-3.0 in 2005 when the project began, reflecting the open-science values of the time. The transition to the MIT License was undertaken to better align with licensing models used across other Galaxy-affiliated projects and to simplify reuse and redistribution of Galaxy components. In particular, this change enables broader downstream distribution of Galaxy and its associated software libraries by communities such as Debian [28], whose packaging guidelines are not fully compatible with AFL-3.0.

## Future directions

Future technical development will continue to emphasize user interface consolidation, expanded workflow documentation standards, enhanced interoperability with research data management systems, and continued modernization of visualization components. These efforts aim to sustain Galaxy as an accessible, reproducible, and extensible platform for global scientific research.

Ongoing and future work focuses on refinement of the upload and data ingestion experience, addressing the increasing complexity of modern data structures, large cohort datasets, and heterogeneous archive formats. A redesigned upload interface will provide clearer feedback, improved collection construction guidance, and more intuitive handling of structured and nested datasets, reducing friction when preparing complex analyses.

Galaxy servers are beginning to add support for user-defined tools, enabling advanced users and domain communities to define, configure, and deploy custom analysis components within controlled environments. This direction lowers the barrier for rapid method integration while preserving Galaxy’s provenance tracking and reproducibility model.

AI-specific integration hooks and deeper integration of the Galaxy-MCP project (https://doi.org/10.5281/zenodo.19833847) will enable external AI clients to interact with Galaxy programmatically, while internal conversational agents will support job error interpretation, tool recommendation, and analysis guidance. These capabilities will extend to tool, workflow, and visualization configuration, troubleshooting, and onboarding, with an emphasis on transparency, user control, and clear separation between automated assistance and reproducible computational steps.

Planned enhancements also include dedicated tool landing pages, similar to the workflow and data landing pages, and a more structured representation of tool state within the Galaxy API.

In parallel, the Galaxy community will continue to address the environmental impact of large-scale computational research. Future efforts will focus on improving job scheduling strategies and resource allocation mechanisms across deployments, encouraging more efficient use of compute infrastructure. This includes promoting tools and datatypes that reduce storage footprint and computational overhead, supporting more efficient intermediate formats, and providing guidance to developers and users toward resource-conscious workflow design.

## Data Availability

Galaxy is freely available at https://galaxyproject.org.

## APPENDIX

Corresponding author: Scott Cain (scott@scottcain.net)

Co-corresponding authors: Anton Nekrutenko (anton@nekrut.org), Björn A. Grüning (bjoern.gruening@gmail.com), Michael C. Schatz (mschatz@cs.jhu.edu), Gareth R. Price (g.price@uq.edu.au)

**Contributors (alphabetical)**

Enis Afgan^33^, Najat Amoukou^74^, Rafael Andrade Buono^78^, Mihail Anton^16^, Christophe Antoniewski^77^, Patrick Austin^48^, Ahmed Hamid Awan^33^, Rolf Backofen^4^, Wendi Anne Bacon^55^, Dannon Baker^33^, Arthur Barreau^50^, Bérénice Batut^23^, Matthias Bernt^60^, Daniel Blankenberg^10^, Anthony Bretaudeau^31^, Catherine Joan Bromhead^58^, Richard Burhans^45^, Melissa Burke^5^, Scott Cain^45,*^, Danielle Callan^54^, Martin Cech^8,29^, María Chavero-Díez^6,63^, Haswane Chei^30^, Ying Chen^56^, Diana Chiang^4^, John M Chilton^45^, Tyler James Collins^33^, Frederik Coppens^78^, Nate Coraor^45^, Charles Coulombe^76^, Michael R Crusoe^21^, Fabio Cumbo^10^, Triskell Cumunel^39^, Michael D'Silva^2^, Armin Dadras^4^, Céline Dalle^22^, Khai Dang^38^, John Y Davis^33^, Paul De Geest^78^, Willem de Koning^19^, Giuseppe Defazio^64^, Boris Depoortere^78^, Katherine Do^73^, José Manuel Domínguez Begines^4^, Bert Droesbeke^78^, Erick Nicolas Duarte^56^, Enol Fernández-del-Castillo^15^, Jeremy Fix^7^, Giulio Formenti^56^, Julian Frey4, Melanie Christine Föll^4^, Carol Gauthier^76^, Kilian Gerberding^4^, Franck Giacomoni^41^, Jeremy Goecks^38^, Nuwan A Goonasekera^58^, Nadia Goué^9^, Timothy J Griffin^73^, Björn Andreas Grüning^4^, Aysam Guerler^33^, Yann Guitton^44^, Ove Johan Ragnar Gustafsson^5,58^, Kristína Gömöryová^36^, Magdalena Harakalova^62^, Helge Hecht^36^, Alireza Heidari^4^, Florian Heyl^26^, Jennifer Hillman-Jackson^45^, Saskia Hiltemann^4^, Mina Hojat Ansari^4^, Hans-Rudolf Hotz^25^, Cameron John Hyde^46^, Pierre-Étienne Jacques^76^, Pratik Dilip Jagtap^73^, Jayadev Joshi^10^, Marie Jossé^12^, Khaled Moahmmad Ahmad Jumah^57^, Arash Kadkhodaei Elyaderani^4^, Katarzyna Kamieniecka^57^, Teja Kattenborn^4^, Markus Konkol^1^, Leonid Kostrykin^27^, Natalie Kucher^33^, Anup Kumar^4^, Mira Kuntz^4^, Bradley William Langhorst^42^, Delphine Lariviere^45^, Yvan Le Bras^40^, Gildas Le Corguillé^51^, Jean Le Cras^24^, Justin Lee^5,32,46^, Jan Leendertse^4^, Hugo Lefeuvre^35^, Brane Leskošek^70^, Leandro Miguel Liborio^48^, Romane Libouban^31^, Marisa Loach^55^, Jose David Lopez Tabernero^4^, Lucille Lopez-Delisle^68^, Daniel Lusk^4^, Alexandru Mahmoud^37^, Molène Mahé^39^, Wolfgang Maier^4^, Igor Makunin^59^, Kirsty McCaffrey^56^, Jack A Medico^56^, Subina Mehta^73^, Hailiang Mei^34^, Mirela Minkova^52^, Saim Momin^4^, Paulo Cilas Morais Lyra Junior^38^, Teresa Müller^4^, Amirhossein Naghsh Nilchi^4^, Tannistha Nandi^66^, Engy Mohamed Taha Nasr^4^, Anton Nekrutenko^45^, Tiffanie Nelson^5,58^, Johannes Nussbaum^53^, Asime James Oba^71^, Łukasz Opioła^3^, Kevin Payet^39^, Melanie Petera^41^, Polina V Polunina^4^, Sergei Pond^54^, Krzysztof Poterlowicz^57^, Gareth Robert Price^5,58,59^, Junhao Qiu^38^, Helena Rasche^34^, Bryan Raubenolt^10^, Tristan N Reynolds^58^, Dave Rogers^11^, Karl Rohr^27^, Gabriel Ferreira Saudade^4^, Michelle Terese Savage^33^, Volodymyr Savchenko^14^, Michael C. Schatz^33^, Isabelle Schmitz^61^, Daniela Schneider^4^, Pauline Seguineau^39^, Beatriz Serrano-Solano^17^, Clea Siguret^23^, Patrik Smeds^45^, Marco Sollitto^56,67^, Nicola Soranzo^13^, Sanjay Kumar Srikakulam^20^, Lieven Sterck^49^, Nikolaos Strepis^19^, Andrew Stubbs^19^, Keith Suderman^33^, Anna Syme^58^, Marco Antonio Tangaro^28^, Reyhaneh Tavakoli Koopaei^69^, Jonathan Andrew Tedds^16^, Mehmet Tekman^4^, Wai Cheng (Mike) Thang^59^, Anil S Thanki^18^, Michael Uhl^4^, Janusch Vajna-Jehle^4^, Marius van den Beek^45,47^, Deepti Varshney^4^, Nikolay Alexandrov Vazov^75^, Jennifer Vessio^33^, Pavankumar Videm^4^, Tomas Vondrak^8^, Reid Wagner^73^, Gregory R Watson^43^, Ralf J. M. Weber^65^, Natalie Whitaker-Allen^33^, Federico Zambelli^72^, Paul Zierep^4^, Rand Zoabi^4^

**Affiliations**

1. 52°North Spatial Information Research, North Rhine-Westphalia 48147, Germany

2. AARNet, Collingwood VIC 3066, Australia

3. Academic Computer Centre CYFRONET of the AGH University of Kraków, Kraków 30-950, Poland

4. Albert-Ludwigs-Universität Freiburg, Baden Württemberg 79110, Germany 

5. Australian Biocommons, Melbourne, Victoria 3052, Australia

6. Barcelona Supercomputing Center, Catalonia 08902, Spain

7. CentraleSupelec, Metz 57070, France 

8. CESNET, Prague 16000, Czechia

9. Clermont Auvergne University, Auvergne-Rhône-Alpes 63000, France

10. Cleveland Clinic, Ohio 44106, United States 

11. Clever Canary, Santa Cruz California 95051, United States

12. CNRS—Data Terra, Rennes 29200, France

13. Earlham Institute, Norwich NR4 7UZ, United Kingdom

14. Ecole Polytechnique Fédérale de Lausanne (EPFL), Vaud 1015, Switzerland

15. EGI Foundation, Amsterdam 1098 XG, The Netherlands

16. ELIXIR, Cambridgeshire CB10 1SD, United Kingdom

17. EMBL, Baden-Württemberg 69117, Germany

18. EMBL's European Bioinformatics Institute (EMBL-EBI), Cambridgeshire CB10 1SD, United Kingdom

19. Erasmus MC, Rotterdam 3015 GD, The Netherlands

20. Forschungszentrum Jülich, Bielefeld 33501, Germany

21. Freie Universität Berlin, Berlin 14195, Germany

22. French Armed Forces Biomedical Research Institute, Brétigny-sur-Orge 91220, France

23. French Institute of Bioinformatics, Ile-de-France 94800, France

24. French Museum of Natural History / Concarneau marine station, Concarneau 29900, France

25. Friedrich Miescher Institute for Biomedical Research, Basel-Stadt 4058, Switzerland

26. German Cancer Research Center, Baden-Württemberg 69120, Germany

27. Heidelberg University, Baden-Württemberg 69120, Germany

28. Institute of Biomembranes, Bioenergetics and Molecular Biotechnologies, National Research Council (CNR), Apulia 70126, Italy

29. Institute of Organic Chemistry and Biochemistry, Prague 16000, Czechia

30. Ionesco, Issy-les-Moulineaux 92130, France 

31. IRISA, Rennes 35042, France

32. James Cook University, Queensland 4814, Australia 

33. Johns Hopkins University, Maryland 21218, United States 

34. Leids Universitair Medisch Centrum, Leiden 2333 ZA, The Netherlands

35. LS2N, Nantes 44300, France

36. Masaryk University, South Moravian 60200, Czechia 

37. Mass General Brigham, Boston Massachusetts 02115, United States

38. Moffitt Cancer Center, Florida 33612, United States

39. Museum national d'Histoire naturelle, Rennes 29900, France

40. Muséum national d'histoire naturelle Station marine de Concarneau, Concarneau 29900, France

41. National Research Institute for Agriculture, Food and Environment (INRAE), Clermont-Ferrand 63000, France

42. New England Biolabs, Ipswich Massachusetts 01938, United States

43. Oak Ridge National Laboratory, Tennessee 37380, United States

44. ONIRIS, Nantes 44307, France 

45. Pennsylvania State University, Pennsylvania 16802, United States

46. Queensland Cyber Infrastructure Foundation, Queensland 4072, Australia

47. SCI-SCALE CommV, Antwerp 2000, Belgium

48. Science and Technology Facilities Council, Oxfordshire OX11 0QX, United Kingdom

49. Sciensano, Brussels 1050, Belgium

50. Sorbonne Université, Rennes 29900, France 

51. Station Biologique de Roscoff - Sorbonne Université/CNRS, Rennes 29680, France

52. SURF, Utrecht 3511 EP, The Netherlands 

53. Swiss National Data and Service Center for the Humanities, Basel-Stadt 4051, Switzerland

54. Temple University, Philadelphia Pennsylvania 19122, United States

55. The Open University, Buckinghamshire MK7 6AA, United Kingdom

56. The Rockefeller University, New York 10021, United States

57. The University of Bradford, West Yorkshire BD7 1DP, United Kingdom

58. The University of Melbourne, Victoria 3052, Australia

59. The University of Queensland, Queensland 4072, Australia 

60. UFZ Leipzig, Saxony 04318, Germany

61. Univ Rouen Normandie, INSA Rouen Normandie, CNRS, Normandie Univ, PBS UMR 6270, Rouen 76000, France

62. University Medical Center Utrecht, Utrecht 3584CX, The Netherlands

63. University of Barcelona, Barcelona 08034, Spain

64. University of Bari Aldo Moro, Bari 70125, Italy

65. University of Birmingham, Birmingham B15 2TT, United Kingdom

66. University of British Columbia, Vancouver V6T 1Z2, Canada

67. University of Florence, Florence 50019, Italy

68. University of Geneva, Geneva 1211, Switzerland

69. University of Isfahan, Isfahan 81746–73441, Iran

70. University of Ljubljana, Faculty of Medicine, Ljubljana 1000, Slovenia

71. University of Maiduguri, Borno State 600004, Nigeria

72. University of Milan, Lombardy 20133, Italy

73. University of Minnesota, MN 55455, United States

74. University of Montpellier, Savigny-sur-Orge 91600, France

75. University of Oslo, Oslo 0316, Norway

76. Université de Sherbrooke, Sherbrooke J1K 2R1, Canada

77. Université Paris Cité, Île-de-France 75013, France

78. VIB Data Core, VIB Technologies, VIB, Ghent, Belgium
